# A Simplified Ductile Fracture Model for Predicting Ultra-Low Cycle Fatigue of Structural Steels

**DOI:** 10.3390/ma15051663

**Published:** 2022-02-23

**Authors:** Mingming Yu, Xu Xie, Shuailing Li

**Affiliations:** College of Civil Engineering and Architecture, Zhejiang University, Hangzhou 310058, China; 22012079@zju.edu.cn (M.Y.); 11612057@zju.edu.cn (S.L.)

**Keywords:** ductile fracture model, uncoupled model, ultra-low cycle fatigue, nonlinear damage increment, ductile capacity consumption, stress triaxiality

## Abstract

Under strong earthquakes, steel structures are prone to undergoing ultra-low cycle fatigue (ULCF) fracture after sustaining cyclic large-strain loading, leading to severe earthquake-induced damage. Thus, establishing a prediction method for ULCF plays a significant role in the seismic design of steel structures. However, a simple and feasible model for predicting the ULCF life of steel structures has not been recognized yet. Among existing models, the ductile fracture model based on ductility capacity consumption has the advantage of strong adaptability, while the loading history effect in the damage process can also be considered. Nevertheless, such models have too many parameters and are inconvenient for calibration and application. To this end, focusing on the prediction methods for ULCF damage in steel structures, with the fragile parts being in moderate and high stress triaxiality, this paper proposes a simplified uncoupled prediction model that considers the effect of stress triaxiality on damage and introduces a new historical-effect related variable function reducing the calibration work of model parameters. Finally, cyclic loading test results of circular notched specimens verify that the proposed model has the advantages of a small dispersion of parameters for calibration, being handy for application, and possessing reliable results, providing a prediction method for ULCF damage of structural steels.

## 1. Introduction

Steel structures are widely used in engineering structures due to their high strength, light weight, and good ductility; however, under strong earthquakes, steel structures will encounter fracture damage due to the crack initiation of ultra-low cycle fatigue (ULCF) [1,2]. The ULCF of structural steel occurs under cyclic large plastic strain loading, and the corresponding fatigue life can generally be considered to be less than 20 cycles [3,4], which is largely different from low cycle fatigue (LCF) and high cycle fatigue. According to experimental results, Kuwamura et al. [5] divided the ULCF failure process of steel structures into three stages, namely the initiation of ductile crack (fiber crack), the steady growth of ductile crack, and the sudden expansion of brittle crack (cleavage failure). The studies [6] found that the initiation of ductile crack accounts for a large part of the overall fatigue life, that is, when the crack appears, it will propagate rapidly under continuous ULCF loading. Therefore, accurate evaluation on the resistance of the structural steel to ULCF damage is of highly significant meaning for the seismic design of steel structures.

So far, many researchers have conducted considerable theoretical studies and experiments on ULCF and proposed some ULCF prediction methods, such as the Gurson-Tvergaard-Needleman (GTN) model [7,8], continuous damage mechanics (CDM) model [9,10], the Coffin–Masson formula [11,12], the cyclic void growth model (CVGM) [13,14], and the ductile fracture model considering the loading history effect [15,16]. These methods can be divided into coupled and uncoupled models [17] according to whether the effect of damage on constitutive properties of materials during the loading process is considered or not. The coupled models couple the constitutive properties with internal damage variables at the material level to describe the continuous degradation of stress or stiffness before cracking, such as the GTN and CDM models. Although the coupled model may reflect the failure process of materials, there are too many parameters in these models, resulting in highly complicated parameter calibration and calculation work.

Conversely, the uncoupled models assume that the evolution of internal damage does not affect constitutive properties of materials before fracture initiation, and it is directly deemed that the material encounters ULCF crack initiation when the damage variable accumulates to a critical value. Due to the advantages of high computational efficiency and the employment of the most accurate uncoupled constitutive models, uncoupled models have been well developed and received widespread attention, such as the Coffin-Manson formula, CVGM, and the ductile fracture model considering the loading history effect. Among them, early studies observed that the Coffin–Masson formula can aptly predict the LCF life of metals but provides an unconservative fatigue life when applied in the range of ULCF [18,19,20]. Therefore, some specific modifications of the Coffin–Masson formula have been conducted by previous researchers to evaluate the ULCF life. XUE introduced an exponential function to make the Coffin–Masson formula suitable for the LCF and ULCF regimes together [21], and Pereira et al. replaced uniaxial equivalent plastic strain with multiaxial strain to consider the effect of the multiaxial stress state [17,22]. Besides, the stress triaxiality related function was introduced into the Coffin–Masson formula by Li et al. [23]. To some extent, these modifications partly promoted the formula’s capability in predicting ULCF. However, specimens with a different notch radius are needed for parameter calibration to consider the multiaxial stress state, and these models cannot reflect the accumulation process of ULCF damage in cyclic loading either. The cyclic void growth model (CVGM) [15] linearly extended the Rice and Tracey ductile fracture model [24] to the cyclic loading case. Regardless of the fact the CVGM shows fairly accurate results in predicting ULCF, it is limited by some assumptions stated in the study. Myers et al. [25] and Yin et al. [26] modified the equivalent plastic strain accumulated in compressive loading as a new damage variable to extend its scope of application on unequal tensile and compressive loading cases, but the studies [27,28,29] show that a problem exists whereby the results predicted by CVGM may occur with large discreteness.

The ductile fracture model for ULCF proposed by Bai et al. [15] based on the ductility capacity consumption method can consider the loading history process by introducing the nonlinear and historical effect functions under the complex loading history and has been shown to have good applicability at the material and component levels [6,30]. The simplified ductile fracture model proposed by Jia et al. [31] adopted the conventional linear evolution hypothesis to calculate damage in the loading process, but it was proved to produce conservative life evaluation under cyclic constant-amplitude loading [32].

Among uncoupled models, the ductile fracture model proposed by Bai et al. involves the effect of the loading history process on evaluating ULCF damage under a full stress state (containing Lode angle parameter and stress triaxiality), which can calculate the ULCF damage of materials in the loading process and has the prospect for further development and application. However, there are too many parameters in this existing model, and the historical effect variable related to the integral of the current and back-stress tensor is difficult to obtain as a whole.

It was reported by many researchers that the ULCF in steel bridge structures mostly occurs at the corner of the column–base plate welded connection where stress and strain concentrations provide a ductile inducement to fracture [33,34,35]. It also occurs at the corner of the steel bridge piers’ base, whose trial-calculated Lode angle parameter is close to 1 or −1 and its stress triaxiality lies in the moderate and high range, which means that the effect of the Lode angle parameter on ULCF damage is weak and can then be ignored [36].

Thence, based on this characteristic of ULCF damage of steel bridge piers, this study dismisses the effect of the Lode angle parameter on damage and introduces a new historical-effect related variable that can be accessed easily and adds the concept of a stress triaxiality cut-off value to achieve the purpose of simplifying and improving the ductile fracture model. To verify the effectiveness of the proposed method, a comparison between the cyclic loading test results of the circular notched specimens, whose stress state is similar to that of the corner point of steel bridge piers, and the predicted results is conducted, showing that the proposed model possesses good accuracy and has a small dispersion of calibrated parameters, thus the number of tested specimens for parameters calibration is reduced. While comparing the proposed ductile fracture model with the linear damage model and the CVGM, we find that the proposed simplified model in this paper has the advantages of possessing good prediction accuracy and being more convenient to calibrate model parameters.

## 2. Simplified Ductile Fracture Model for ULCF

The method in this paper is based on the ductile fracture model to calculate the ULCF damage accumulated in the process of cyclic large strain loading. Furthermore, the ductile fracture model proposed by Bai et al. [30] can consider the ULCF of metal materials under a full stress state including varied Lode angle parameters and stress triaxiality. The expression is shown in the following Equation (1):

(1) $$dD=g\left(D\right)\cdot h\left(D,\mu \right)\frac{d\varepsilon_{p}}{\varepsilon_{f}\left(T,\bar{\theta }\right)}$$

where d*D* is the damage index increment, g(D) is the nonlinear function expressed in Equation (2) that plays an important role in calculating the damage index in a nonlinear manner, as the equivalent plastic strain $\varepsilon_{p}$ increases; and h(D,μ) shown in Equation (3) represents the historical effect function, which considers the effect of the direction change in the non-proportional loading between the current stress and the back-stress tensor. Equation (1) assumes that these two functions, g(D) and h(D,μ), act independently and simultaneously throughout the entire loading process. $T=\sigma_{eq}/\sigma_{m}$ is the dimensionless stress triaxiality, wherein $\sigma_{eq}$ is the hydrostatic pressure and $\sigma_{m}$ is the Mises stress. $\bar{\theta }$ denotes the Lode angle parameter whose detailed expression can refer to [37], $\varepsilon_{f}\left(T,\bar{\theta }\right)$ is the fracture equivalent plastic strain related to T and $\bar{\theta }$, and $d\varepsilon_{p}=\sqrt{\left(2/3\right)d\varepsilon_{ij}^{p}\cdot d\varepsilon_{ij}^{p}}$ represents the equivalent plastic strain increment.

(2) $$g\left(D\right)=\left(c_{g}D+\frac{c_{g}}{e^{c_{g}}-1}\right)$$

(3) $$h\left(D,\mu \right)={\left(1+c_{h}D^{\beta_{1}}\mu^{\beta_{2}}\right)}^{k}$$

Specifically, in Equations (2) and (3), $c_{g}$ is the control parameter, and μ is a scalar variable that can capture the influence of the key source of the loading path change, whose detail expression is referred to in [30]. Model parameters such as $c_{h}$, $\beta_{1}$, $\beta_{2}$, *k* are calibrated by experiment to better adjust for the prediction.

Due to the historical-effect-related part, Equation (3) has four parameters to calibrate, and the form of that is also complicated, resulting in the mutual coupling effect among the model parameters, which will lead to inconvenience in calibration and application. Therefore, this paper simplifies it by introducing a new plain function $r\left(D,\varepsilon_{acc}\right)$ as shown in Equations (4) and (5), related to $\varepsilon_{acc}$, which is the cumulative equivalent plastic strain under compressive loading. Meanwhile, the proposed model also introduces the concept of the stress triaxiality cut-off zone shown in Equation (4), which is considered to have a large impact on controlling the ductile damage [16,38,39]. The damage index increment will be taken into account when the stress triaxiality is larger than the value of −1/3; otherwise, we assume that it does not affect the ductile damage.

(4) $$dD=\left\{ \begin{array}{l} r\left(D,\varepsilon_{acc}\right)\cdot \frac{d\varepsilon_{p}}{\varepsilon_{f}\left(T\right)}\;\;\;T\geq -1/3\\ 0\;\;\;\;\;\;\;\;\;\;T<-1/3 \end{array}\right.$$

(5) $$r\left(D,\varepsilon_{acc}\right)=\left(AD+\frac{A}{e^{A}-1}\right)e^{\left(B\varepsilon_{acc}-C\right)}$$

In Equations (4) and (5), $r\left(D,\varepsilon_{acc}\right)$ is the function that captures the nonlinearity and historical effect in the loading process, and $\varepsilon_{acc}=\sum_{\mathrm{compressive}}\int_{\varepsilon_{1}}^{\varepsilon_{2}}d\varepsilon_{p}$ represents the equivalent plastic strain accumulated under compressive loading. *A*, *B*, *C* are model parameters related to materials, and $\varepsilon_{f}\left(T\right)$ is the equivalent plastic strain at fracture related to stress triaxiality only.

The explanation for such simplicity in the historical effect function is that $\varepsilon_{acc}$ is also a scalar variable, which will grow in a monotonous manner, like μ, with the increase of the equivalent plastic strain under cyclic loading [16,30]; thus, the influence of cyclic loading can also be captured similarly, and the rationality can be realized in the next validation work.

There are some statements about the stress triaxiality cut-off value and the reason we chose the value of −1/3. Bao et al. [38] proposed the concept of stress triaxiality cut-off value considered to be −1/3 constantly, but Khan and Liu (2012a) [40] reported that AA 2024-T351 under non-proportional biaxial compression conditions fractured in ductile manner with a stress triaxiality of −0.497, which makes us think that setting the stress triaxiality cut-off value as −1/3 constantly may be irrational. Wen et al. [16], whose work was based on the Cockcroft–Latham–Oh criterion [41,42], and other scholars [37,43] have conducted related discussions and research, but in general, on the basis of existing experimental techniques, it is still a big challenge to accurately determine the stress triaxiality cut-off value of metal materials and the effect of the Lode angle parameter. Therefore, the stress triaxiality cut-off value is introduced as −1/3 constantly for simplification here, which corresponds to the research of Wen et al. under moderate- and high-stress triaxiality.

Meanwhile, we modify the fracture equivalent plastic strain from the modified Mohr–Coulomb formula [37] in the initial model to the stress-modified critical strain model (SMCS) [44] expressed in Equation (6) to dismiss the effect of the Lode angle parameter on damage, due to the value of the corner points of steel piers base being close to −1 or 1, to further simplify the model parameters.

(6) $$\varepsilon_{f}\left(T\right)=\alpha \exp\left(-1.5T\right)$$

where α is the ductility coefficient that needs to be calibrated by monotonic loading tests.

**Figure 1:**
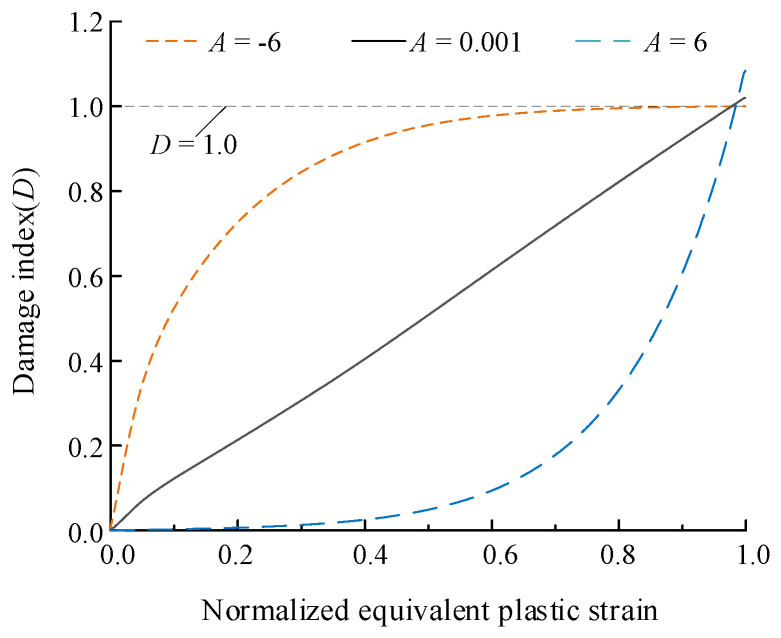
Damage index (D) accumulated.

It shows that when the *A* variable changes from a negative value to a positive value, the damage accumulation behavior increases from a convex function to a concave form, as shown in Figure 1, in which normalized equivalent plastic strain represents $\varepsilon_{p}$ divided by the max value of that at fracture. If *A* takes a very small positive value, the damage can be reset to behave linearly. When *B* takes a positive value, it is assumed that the material will undergo plastic strengthening during the cyclic loading process, but on the contrary, it is understood that plastic softening occurs. Besides, *C* as a ductility parameter controls the overall ductility capacity of materials, and the ductility is better with a larger value of *C*. The comprehensive calibration of the parameters will be carried out in Section 4.

When we do not consider the nonlinear and historical effect on damage, Equation (4) can degenerate into Equation (7), which adopts the linear damage evolution.

(7) $$dD=\left\{ \begin{array}{l} \frac{d\varepsilon_{p}}{\varepsilon_{f}\left(T\right)}\;\;\;T\geq -1/3\\ 0\;\;\;\;\;\;T<-1/3 \end{array}\right.$$

## 3. Cyclic Loading Tests of Circular Notched Specimens

**Figure 2:**
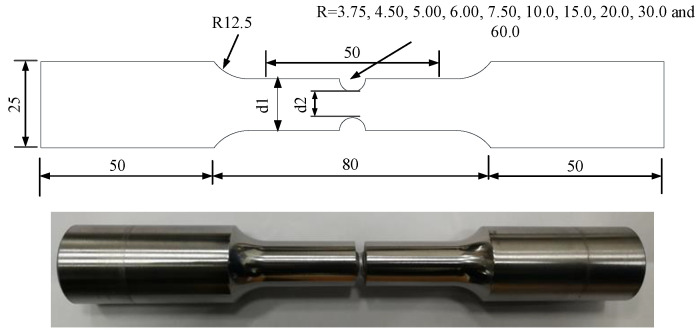
The structure and dimensions of the circular notched specimen (unit: mm) [23].

In order to verify the performance of the proposed model in predicting the ULCF of steel bridge piers, we selected constant-amplitude cyclic large strain loading test results of circular notched specimens with varieties of notch radii whose stress state is similar to that of the corner point of a steel bridge pier for comparison from [23,45] and briefly state the related information. The structure and size of the circular notched specimens are shown in Figure 2. The gauge length of the extensometer is 50mm, which is used to control the loading strain range and rate during the tests. Besides, the cyclic loading tests were carried out using MTS 880 (MTS System Corporation, Eden Prairie, MN, USA).

The mechanical characteristic parameters of Q345qC steel, which is widely used in steel bridges in China, can be seen in Table 1.

## 4. Calibration Work of Model Parameters

### 4.1. Establishment of Finite Element Model

**Figure 3:**
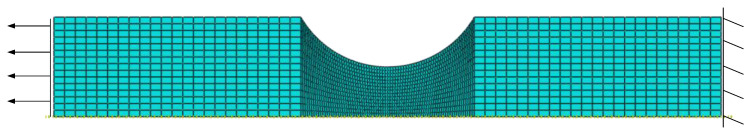
The FEA model of the gauge length part.

Due to the fact the loading procedure containing the strain range and rate is controlled by the gauge length part, and that some previous researchers [32,46] have shown that the simulation result of modeling only the gauge part is almost identical to that of the overall specimen, only the finite element model of the gauge length part is established here for simplicity, in order to obtain the related information needed in the proposed model. The two-dimensional axisymmetric finite element model in ABAQUS 6.14-4 (Dassault Systèmes Simulia Corporation, Paris, France) is used to consider the axial symmetry of specimens’ structure and loading program as shown in Figure 3, and the reduced integration element (CAX8R) was applied. We encrypt the elements near the notch by adopting an element size of approximately 0.2 mm, which is similar to the characteristic length of the Q345qC steel used [47], while in the other part, sparse grid sizes were adopted at approximately 0.5 mm.

The Lemaitre-Chaboche hybrid hardening model [48,49] is used to simulate the cyclic plasticity behavior, including isotropic and kinematic hardening. Here, the calibrated true stress–plastic strain curve is adopted to simulate kinematic hardening, which can be realized in ABAQUS 6.14 by importing true stress-plastic strain data in the half-cycle option of the plasticity module. The isotropic hardening is explicitly described by the following Equation (8):

(8) $$\sigma^{0}=\sigma_{0}+Q_{\infty }\left[1-\exp\left(-b\varepsilon_{p}\right)\right]$$

where $\sigma_{0}$ represents the yield stress at zero plastic strain, $Q_{\infty }$ represents the maximum change value of the yield surface, and *b* is the rate at which the size of the yield surface changes with the development of plastic strain.

**Figure 4:**
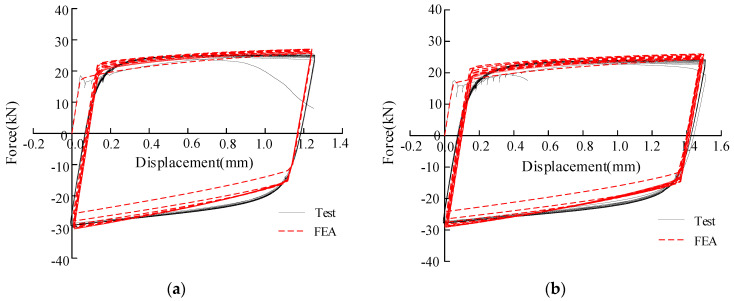
Comparison of force-displacement curves of specimens obtained by tests and FEA: (**a**) SP-142; (**b**) SP-176.

It can be seen that the good fit of the force-displacement curves before the curvature mutation point of SP-142 and SP-76 selected from [23] means the established FEM model and simulation of the cyclic plasticity hardening for Q345qC is satisfactory, as shown in Figure 4, in which force is the reactive force in the fixed end and displacement denotes the relative displacement between both ends of the gauge length part. Only the compressive part and the Bauschinger effect simulation are slightly offset. However, we assume that most of the compressive loading will not affect the ductile damage due to the existence of the stress triaxiality cut-off zone mentioned before, while the simulation of the tensile part is highly identical to the experimental result, which mainly affects the evolution of ULCF damage. Other specimens that are not shown here have similar trends as well. This demonstrates the rationality of the finite element model and constitutive parameter selection, which guarantees we can calibrate the model parameters and predict damage relatively accurately in the next step.

### 4.2. Calibration of Model Parameters

Four parameters need to be calibrated for the proposed model in this paper; among them, α in $\varepsilon_{f}\left(T\right)$, shown in Equation (6), is obtained by monotonic tensile tests, here referring to the value α = 2.07 [50], while the other three parameters are acquired through cyclic loading tests of circular notched specimens. Here, we take the sudden change point of the slope of the load–displacement curve as the fracture initiation point [13], which is input as the key displacement into the proposed model for the parameter calibration and corresponding verification.

Firstly, finite element simulations were conducted until the fracture initiation point of the test specimens without involving any fracture option for calibration, so that the model related data under cyclic loading such as stress triaxiality *T*, equivalent plastic strain $\varepsilon_{p}$, and historical-effect related parameters $\varepsilon_{acc}$ are extracted. Then, a Matlab code was created to determine the approximate parameter range and calculate the damage evolution integration in Equation (4), in order to obtain a set of parameters that ensure the calculated damage index at the fracture initiation point for the calibrated tests is as close to unity as possible. In addition, the average error between the experiment and predicted ULCF life should be as small as possible, referring to Equation (9) for evaluation, where $N_{\exp}^{i}$ represents the experiment life of the specimen whose number is *i*, and similarly, $N_{\mathrm{FEA}}^{i}$ denotes the predicted ULCF life of the corresponding specimen, while *N* is the total number of specimens used for calibration:

(9) $$Error=\min\left[\left(\sum_{i=1}^{N}\frac{\left|N_{\exp}^{i}-N_{\mathrm{FEA}}^{i}\right|}{N_{\exp}^{i}}\right)/N\right]$$

In order to examine the discretion performance of the three parameters, *A*, *B*, and *C*, in the proposed model, we divide the specimens selected from [23,45] into eight groups, which include as many different notch radii or loading procedures as possible to make the work more convincing; the detailed grouping information is not displayed here. The calibration results are shown in Table 2.

**Figure 5:**
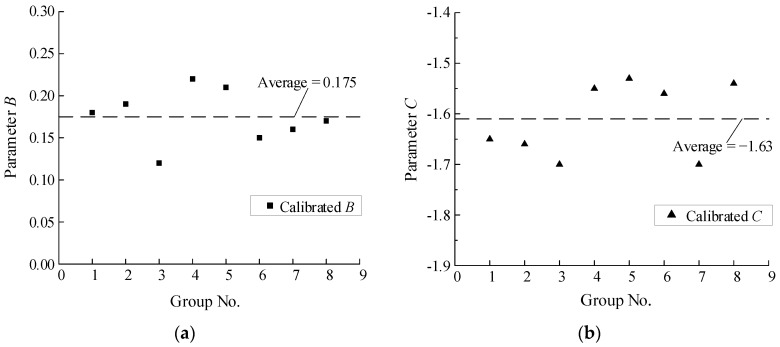
The distribution of parameter value: (**a**) Parameter *B*; (**b**) Parameter *C*.

The average value of *A* is −6 constantly, which may be considered irrelevant to the material but is still considered to be related to the materials here and will provide a reference for other metal materials; in addition, this value is identical to that of Inconel 718 as performed in [30]. The average values of *B* and *C* are 0.175 and −1.61, respectively, and we consider these two parameters as materials related to the relatively small dispersion. While the dispersion of parameter *B* is comparatively larger than that of parameter *C*, further investigation found that the damage is more sensitive to the change in parameter *C*, which indirectly reflects that the calibration of these three parameters is reasonable. The discretion distribution of parameters *B* and *C* can be concretely shown in Figure 5, and that of *A* is not displayed here for the fact the discretion of parameter *A* is none.

Referring to the above-obtained results, we find that the model parameters *A*, *B*, and *C* have a relatively high correlation with the materials. Therefore, for the sake of making the calibration simpler, reasonable, feasible, and easy for operation, five specimens, whose detailed information is shown in Table 3, are used for the model parameter calibration.

**Figure 6:**
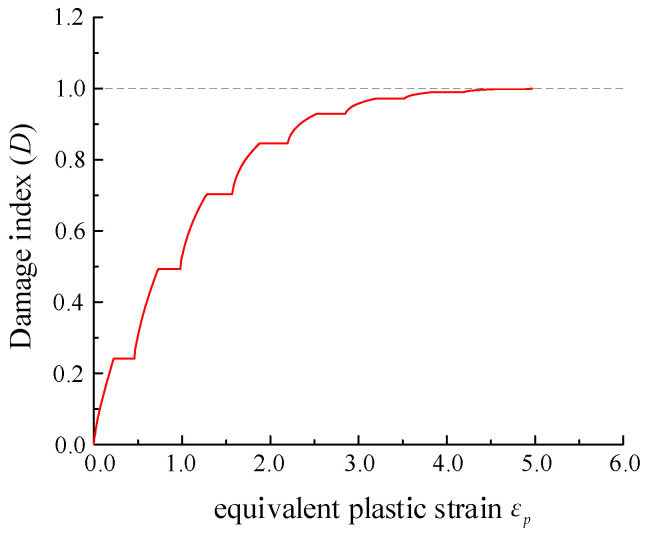
Damage index related to equivalent plastic strain $\varepsilon_{p}$.

The calibrated parameter *A*’ value is −6, *B* equals 0.18, and *C* is −1.65; additionally, their corresponding discrepancy with the average value is small, so it indirectly reflects the fact the simplified calibration work is certainly convincing. Figure 6 shows the damage evolution of a selected specimen calculated by the obtained parameters, illustrating the nonlinearity of damage in the loading process.

## 5. The Validation of the Proposed Model

### 5.1. Evaluation of Prediction Accuracy

Table 4 shows the specimens for validation from [23,45] and the concrete information about them, such as the predicted life and relative error, which are calculated using the calibrated parameters. The comparison of the experiment life and ULCF results predicted by the proposed model can be seen in the following section, and relative error γ is introduced according to Equation (10) in order to evaluate the prediction accuracy of the proposed model.

(10) $$\gamma =\frac{\left|N_{\exp}-N_{\mathrm{FEA}}\right|}{N_{\exp}}$$

The proposed model shows good performance in its predictive ability and corking operability for the reason that only five tests are used for parameter calibration, which reduces the workload required, and the average relative error of prediction is 12.95%, indicating that the results are promising and reliable.

Owing to this validation, mainly for the specimens whose fatigue life is less than 20 cycles, that is, the ULCF regime on which the proposed model focuses, the predicted results are relatively satisfactory. However, the reason that the error for some specimens exceeds 20% can be explained by the fact that when the fatigue life is short, besides, the experiment may sometimes produce accidental deviation even though the prediction results obtained are close to the experimental results, the relative percentage error will appear to be relatively large. Promisingly, when the fatigue life is between 10 and 20 cycles, it shows much better prediction results, and the error is mostly within the 15% line. Besides, it can also be seen that when the fatigue life is relatively large (more than 20 cycles), which means the loading strain is small, the deviation between the experimental and the predicted results increases, showing the trend of conservative prediction, which may be favorable for a safe objective. What causes this phenomenon may be the existence of a different crack or void growth/coalescence behavior in ULCF or other low-cycle fatigue mechanisms that play a role in these experiments. However, most of the points in the comparison between the predicted results and the experiment life are within the 20% error line on the whole, which demonstrates that the proposed model is promising and capable of predicting ULCF failure of Q345qC steel under moderate- and high-stress triaxiality, and can be further adopted in predicting ULCF behavior of the corner point of steel bridge piers.

### 5.2. Comparison with Other Models

#### 5.2.1. Comparing with Lining Damage Model

**Figure 7:**
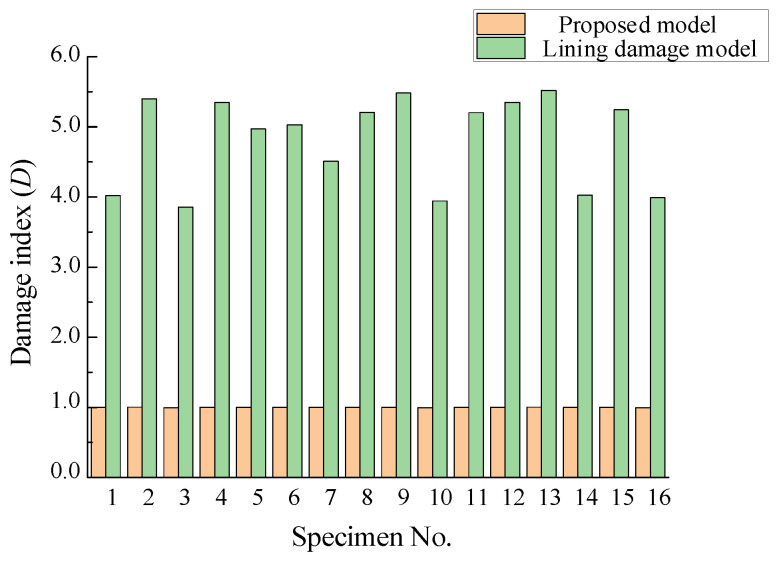
Comparison of damage index for proposed model and lining damage model.

We mentioned before that it is inaccurate to consider only the linear incremental damage [32] of the material as shown in Equation (7), and the nonlinear and historical effect under cyclic constant strain loading is significant in the ductile fracture model. Here we will illustrate the statements vividly by comparing the damage index calculated by Equation (7) and the proposed model until the initiation of the fracture placement of the selected specimens, as shown in Figure 7.

It can be seen that the damage index calculated by Equation (7) is greater than unity, and almost it exceeds by a great deal, which will make the prediction very conservative. In contrast, that value of the proposed model is close to 1 on the whole, and in actuality, the corking prediction ability of the proposed model has been shown in the previous section. Therefore, the comparison of these two groups clearly shows the influence of the nonlinearity and historical loading effect on the damage under large strain cyclic loading.

#### 5.2.2. Comparison with CVGM

This part focuses on comparing the features and predicted results of the proposed model with those of CVGM, as the CVGM is widely used in ULCF life prediction and belongs to an uncoupled model as well. The expression of the CVGM can be seen in Equations (11) and (12). The cyclic void growth index $VGI_{cyclic}$ representing the cyclic void growth demand is defined in Equation (11) and the critical void growth capacity under cyclic loading $VGI_{cyclic}^{critical}$ is described in Equation (12) referring to [13], where $\varepsilon_{1}$ and $\varepsilon_{2}$ represent the equivalent plastic strain at the beginning and end in each tensile or compressive loading cycle, respectively, and $VGI_{mono}^{critical}$ is the monotonic void growth capacity expressed in $VGI_{mono}^{critical}=\int_{0}^{\varepsilon_{p}^{critical}}\exp\left(1.5T\right)d\varepsilon_{p}$, where $\varepsilon_{p}^{critical}$ is the critical fracture strain under monotonic loading. f is a degraded function related to $\varepsilon_{acc}$, and λ indicates the cyclic damage degradation parameter.

(11) $$VGI_{cyclic}=\sum_{T\geq 0}\int_{\varepsilon_{1}}^{\varepsilon_{2}}\exp\left(\left|1.5T\right|\right)d\varepsilon_{p}-\sum_{T<0}\int_{\varepsilon_{1}}^{\varepsilon_{2}}\exp\left(\left|1.5T\right|\right)d\varepsilon_{p}$$

(12) $$VGI_{cyclic}^{critical}=VGI_{mono}^{critical}\times f=VGI_{mono}^{critical}\times \exp\left(-\lambda \varepsilon_{acc}\right)$$

**Figure 8:**
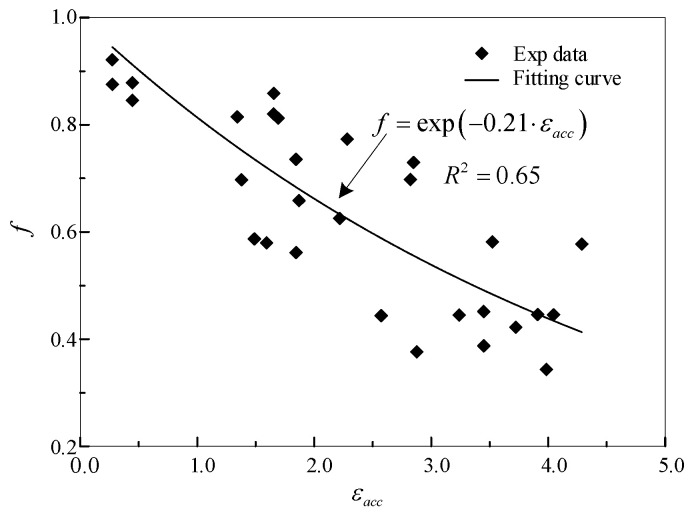
The fitting curve of parameter λ in CVGM.

The parameters in CVGM obtained here are $VGI_{mono}^{critical}=2.03$ [45], λ=−0.21, λ is calibrated as shown in Figure 8, and Exp denotes the experimental data.

One thing that needs to be noted is that the CVGM degradation parameters of Q345qC steel have been calibrated before [45], and λ is calibrated to 0.12, which is highly deviated from the value obtained here, due to the fact that the damage variable $\varepsilon_{acc}$ in the CVGM used here replaces $\varepsilon_{p}^{accumulated}$, which represents the equivalent plastic strain accumulated at the starting point of the latest tensile loading in the original CVGM. Besides, the damage variables used here have been proved to be more suitable than that of the original model in many cases, such as the situation where the tensile part is larger than that of the compressive part [25,26], which will mean the model parameters obtained in this way are of more referential significance.

**Figure 9:**
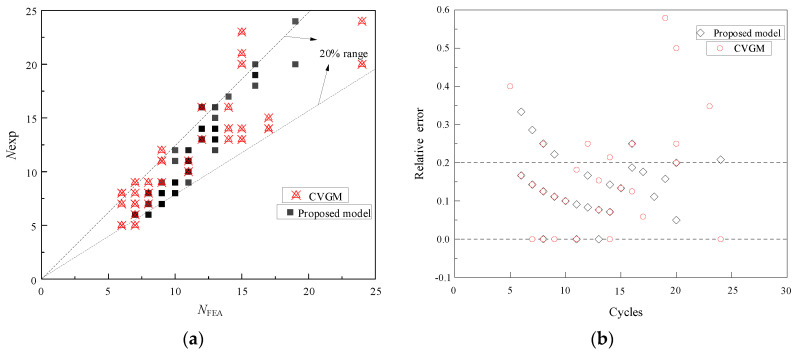
Comparison with CVGM: (**a**) Prediction ability; (**b**) relative error.

Furthermore, the prediction results and error comparison of the proposed model and the CVGM are shown in Figure 9. They illustrate that the results predicted by the proposed model are relatively concentrated, most of which are distributed within the 20% error line, while there are more specimens with a prediction error of more than 20% in the CVGM, and the average relative error calculated by the proposed model is 12.95%, which is a certain improvement over 14.96% of the CVGM. In general, regardless of the discretion of model parameters or error point distributions, the proposed model both performs better than CVGM and shows stability and reliability in predicting the ULCF life.

## 6. Conclusions

In this paper, a simplified ductile fracture model is proposed on the basis of the ULCF characteristic of steel bridge piers, and cyclic loading tests of Q345qC steel, which is commonly used in steel bridge structures in China, are selected for calibration and validation. A simple finite element model is established for obtaining the related information needed in the ULCF damage prediction process, then the parameter dispersion and the actual calibration work of the proposed model are analyzed. Finally, the prediction performance is demonstrated based on the fatigue test results of circular notched specimens, and a comparison with the linear damage model and CVGM was conducted. Based on the above-conducted research, the following conclusions can be drawn:

(1) The proposed model has fewer parameters needed for calibration when compared with the original model, and the coupling between the parameters is reduced. Moreover, the discreteness of the parameters obtained through the grouping calibration verifies that the proposed model is less sensitive to parameter *B* but with larger dispersion, compared to parameter *C*. Therefore, using fewer test specimens to calibrate the required parameters is possible in the proposed model, which means obtaining parameters is more convenient and shows high reliability as well.

(2) The predicted life is very close to that of the tests, and the relative error is small when considering the influence of the nonlinear historical parts, which shows the nonlinear and simplified historical effect parts considered in the model can perform well in the ULCF damage prediction.

(3) Compared to the commonly used CVGM model, it is found that although the proposed model has more parameters than the CVGM model, the cyclic damage degradation parameter λ calibrated in the CVGM is more discrete than parameters in the proposed model. Fewer test specimens are needed for the proposed model’s parameter calibration due to the smaller parameter dispersion, while it is the opposite in the CVGM. The average error of prediction results for the proposed model is 12.95%, which performs somewhat better than the CVGM model with 14.96%. Besides, the dispersion of the prediction results by the proposed model is smaller, with higher reliability, and the application of the proposed model is simpler as well.

In summary, the results show that combining the incremental form of ductility consumption under monotonic loading and the nonlinearity and historical effects under cyclic loading is reasonable for calculating the ULCF damage of structural steels. The proposed model performs better than the CVGM under the conditions implemented here and is thought to be a simple and feasible method for predicting ULCF damage. Meanwhile, it is worth exploring whether, when the Lode angle parameter is taken into account, the proposed model can predict the ULCF failure of steel structures under a full stress state, and this may be suggested as a future research path.

## Figures and Tables

**Table 1:** Mechanical properties of Q345qC steel [45].

E(MPa)	$\sigma_{y}\left(\mathrm{MPa}\right)$	$\sigma_{u}\left(\mathrm{MPa}\right)$	$\varepsilon_{f}$	$\sigma_{f}\left(\mathrm{MPa}\right)$	*A_l_* (%)
198,221	351.1	508.57	1.14	1104.57	40.6

Explanation: *E* represents the modulus of elasticity; $\sigma_{y}$ and $\sigma_{u}$ represent the yield strength and ultimate strength, respectively; $\varepsilon_{f}$ and $\sigma_{f}$ represent the true strain and stress at the point of tensile fracture, respectively; *A_l_* represents the section shrinkage rate.

**Table 2:** The value of model parameters.

Group No./Parameters	*A*	*B*	*C*
1	−6	0.18	−1.65
2	−6	0.19	−1.66
3	−6	0.12	−1.7
4	−6	0.22	−1.55
5	−6	0.21	−1.53
6	−6	0.15	−1.56
7	−6	0.16	−1.7
8	−6	0.17	−1.54
Average	−6	0.175	−1.61
COV	0	0.173	0.042

**Table 3:** Specimens used for parameter calibration.

Notch Radius (mm)	No.	*d*_1_ (mm)	*d*_2_ (mm)	Loading Strain	Cycles to Fracture Initiation (*N*_exp_)
3.75	BM-1	15	7.5	[0,1.60%]	7
	BM-2	15	7.5	[0,1.60%]	8
4.5	BM-3	15	7.5	[0,1.35%]	14
	BM-4	15	7.5	[0,1.35%]	13
10	BM-5	15	7.5	[0,2.50%]	7
	BM-6	15	7.5	[0,2.50%]	8
15	BM-7	15	7.5	[0,3.00%]	7
	BM-8	15	7.5	[0,3.00%]	9
30	BM-9	15	7.5	[0,3.00%]	14
	BM-10	15	7.5	[0,3.00%]	14

Note: For example, [0, 3.00%] means the gauge length part of specimens cycled between strain 0 and 3.00%.

**Table 4:** Specimens used for verification and results.

Notch Radius (mm)	Loading Strain	Cycles to Fracture Initiation (*N*_exp_)	*N*_FEA_	Error
3.75	[0, 1.50%]	8	10	25.00%
	[0, 1.50%]	9	10	11.11%
	[0, 1.25%]	14	12	14.29%
	[0, 1.25%]	14	12	14.29%
4.5	[0, 1.10%]	20	16	20.00%
	[0, 1.10%]	19	16	15.79%
	[0, 1.30%]	14	13	7.14%
	[0, 1.30%]	14	13	7.14%
	[0, 1.30%]	15	13	13.33%
	[0, 1.50%]	10	11	10.00%
	[0, 1.50%]	11	11	0.00%
5.0	[0, 1.80%]	8	9	12.50%
	[0, 1.80%]	8	9	12.50%
6.0	[0, 1.50%]	14	13	7.14%
	[0, 1.50%]	14	13	7.14%
	[0, 2.00%]	8	9	12.50%
	[0, 2.00%]	7	9	28.57%
7.5	[0, 3.00%]	6	7	16.67%
	[0, 2.50%]	8	8	0.00%
	[0, 2.50%]	7	8	14.29%
	[0, 2.00%]	11	10	9.09%
	[0, 2.00%]	12	10	16.67%
10	[0, 2.00%]	10	11	10.00%
	[0, 2.00%]	11	11	0.00%
	[0, 1.80%]	13	13	0.00%
	[0, 1.80%]	16	13	18.75%
15	[0, 3.50%]	6	8	33.33%
	[0, 3.50%]	6	8	33.33%
	[0, 2.50%]	12	11	8.33%
	[0, 2.50%]	12	11	8.33%
20	[0, 2.20%]	17	14	17.65%
	[0, 2.50%]	16	12	25.00%
	[0, 2.50%]	13	12	7.69%
	[0, 3.00%]	9	11	22.22%
	[0, 3.00%]	11	11	0.00%
30	[0, 3.50%]	8	10	25.00%
	[0, 3.50%]	9	10	11.11%
60	[0, 2.50%]	20	19	5.00%
	[0, 2.50%]	24	19	20.83%
	[0, 3.00%]	19	16	15.79%
	[0, 3.00%]	18	16	11.11%
	[0, 3.50%]	13	13	0.00%
	[0, 3.50%]	12	13	8.33%
Average				12.95%

Note: For example, [0, 2.50%] means the gauge length part of specimens cycled between strain 0 and 2.50%.

## Data Availability

Not applicable.
